# Winning the endgame against sleeping sickness: Efficacy profiling of acoziborole against African trypanosomes with the focus on *Trypanosoma brucei rhodesiense*

**DOI:** 10.1371/journal.pntd.0014762

**Published:** 2026-10-05

**Authors:** Monica Cal, Sonja Keller, Romina Rocchetti, Stéphanie Braillard, Jean-Robert Ioset, Pascal Mäser, Marcel Kaiser

**Affiliations:** 1 Swiss Tropical and Public Health Institute, Allschwil, Switzerland; 2 University of Basel, Basel, Switzerland; 3 Drugs for Neglected Diseases initiative, Geneva, Switzerland; Umaru Musa Yar'Adua University, NIGERIA

## Abstract

Human African trypanosomiasis (HAT) is caused by two subspecies of *Trypanosoma brucei*, *T. b. gambiense* and *T. b. rhodesiense*, with the former accounting for over 95% of all cases. HAT is an elusive disease, which is fatal unless treated, with a focal prevalence in the rural areas of tropical Africa where the tsetse fly vectors are endemic. At the same time, HAT is one of the very few infectious diseases where global elimination seems possible. The incidence is at a historic low, and new orally bioavailable drugs have been developed that will expand the coverage of treatment in endemic regions. In particular, acoziborole has passed the clinical development as a single-dose oral cure of *T. b. gambiense* infection. However, to win the endgame against HAT, acoziborole also needs to be extended to *T. b. rhodesiense*. In order to strengthen the preclinical dossier of acoziborole, we conducted an in-depth evaluation of acoziborole against the bloodstream forms of all three *T. brucei* subspecies with a focus on *T. b. rhodesiense*. Acoziborole had consistent and high activity against different *T. b. rhodesiense* strains, including a multidrug-resistant field isolate, with IC_50_ values around 600 nM. Acoziborole is faster acting than fexinidazole, and it exhibits irreversible cidality against *T. b. rhodesiense* at concentrations of ≥4.3 μM (1.6 μg/ml) after 15 h of incubation. At very short exposure times (≤8 h), *T. b. gambiense* appeared to be more sensitive than *T. b. rhodesiense*. However, given the extremely long elimination half-life of acoziborole in the human body, this difference will be irrelevant for its trypanocidal action in patients. We therefore conclude that acoziborole is expected to be as effective against *r*-HAT as against *g*-HAT in patient populations.

## Author summary

Human African trypanosomiasis (HAT) is a neglected tropical disease that is found exclusively in sub-Saharan Africa. The causative agents are the parasites *Trypanosoma brucei gambiense* in West and Central Africa, and *T. b. rhodesiense* in East Africa. Once a devastating scourge across tropical Africa, HAT today is under control with fewer than 1000 reported cases per year. This low incidence raises hopes to reach the goal of zero transmission, particularly so since major breakthroughs have been achieved in the development of new, orally applicable drugs: fexinidazole in 2019, and acoziborole in 2026, have obtained positive opinion by the European Medicines Agency for the treatment of HAT. Whereas fexinidazole is registered for both *T. b. rhodesiense* and *T. b. gambiense*, acoziborole is only approved for the latter; the reason is that there are too few cases of *T. b. rhodesiense* to conduct a clinical trial. Yet, even though currently of lower medical importance than *T. b. gambiense*, *T. b. rhodesiense* will be more difficult to control in the long run because it is a zoonotic pathogen. Acoziborole as a single-dose cure would be a tremendous help in the therapy of sporadic cases or outbreaks. Here we provide an in-depth profiling of acoziborole against *T. b. rhodesiense* isolates, demonstrating its full effectivity at the clinically relevant levels. We hope these findings pave the way for the use of acoziborole also against the East-African pathogen *T. b. rhodesiense*.

## Introduction

Human African trypanosomiasis (HAT, also known as sleeping sickness) is a fatal disease with limited treatment options and no vaccine. There are two subspecies of *Trypanosoma brucei* that cause geographically, epidemiologically, and clinically different forms of HAT: *Trypanosoma b. rhodesiense* occurs in eastern and southern Africa, has a large zoonotic reservoir, and causes a fast-progressing disease in humans (*r*-HAT). *Trypanosoma b. gambiense* is endemic to western and central Africa, causes a slowly progressing disease (*g*-HAT), and does not have a significant reservoir in animals. Today, over 95% of all reported cases are due to *T. b. gambiense*. This is why drug development efforts have initially concentrated on *g*-HAT. Drug development typically consists of preclinical research with the non-human-infective subspecies *T. b. brucei*, followed by clinical studies with *T. b. gambiense*. A particular challenge to drug development for HAT is the requirement for trypanocidal molecules to penetrate the blood-brain barrier in order to cure the second stage of the disease, once the trypanosomes have infested the patient's central nervous system.

After decades of inadequate, impractical, and toxic treatment options, drug development for HAT has finally turned into a success story. Fexinidazole has emerged as the first all oral medication for HAT [1–3], effectively replacing older treatments. Fexinidazole is now recommended as first-line treatment for the management of *g*-HAT first and second stage, except for severe cases (≥100 white blood cells per µl of CSF) and children below 6 years of age or 20 kg body weight [4], and *r*-HAT first and second stage, except for children below 6 years or 20 kg [5]. Previously, NECT (nifurtimox-eflornithine combination therapy) and melarsoprol have been used as the standard therapies for late-stage *g*-HAT and *r*-HAT, respectively [4–6]. NECT has a very good efficacy but cannot be used for *r*-HAT, and it is not well-suited for field conditions because it is administered by intravenous infusion and requires hospitalization of the treated patients. Melarsoprol is associated with adverse events not acceptable by modern standards, but it is still the first-line treatment for children <6 years or <20 kg with second stage *r*-HAT. Before the introduction of NECT, melarsoprol had also been used for second stage *g*-HAT. Fexinidazole has a better tolerability and a simpler regimen than all previous drugs. However, fexinidazole still requires dosing for ten consecutive days under direct observation, fed conditions, and eventually a lumbar puncture in the case of suspicion of severe second stage *g*-HAT [7].

The treatment of HAT will be further simplified by the introduction of acoziborole (previously named SCYX-7158 and AN5568), a new chemotype that has passed the final stages of clinical testing for *g*-HAT [8–11]. Once approved for use, acoziborole will be the first oral single-dose cure [12]. Acoziborole has received positive opinion by the European Medicines Agency in February 2026. After fexinidazole, which has been in use for five years now, acoziborole will be the next game changer in the management of HAT, once recommended by the WHO. Its safety and ease of use will further simplify the diagnostic algorithm and increase the coverage of patients who receive effective treatment. This is essential when combatting a disease like HAT, which affects remote rural regions the where tsetse flies are found, and which has a highly focal and locally variable distribution [13].

Thanks to increased surveillance and treatment efforts, combined with vector control programmes (and also loss of tsetse fly natural habitat due to human expansion [14, 15]), the incidence of HAT is at a historic low. Since 2018, the total number of reported HAT cases has dropped below 1000 per year [16]. Thus, the WHO has reached its goal of elimination of HAT as a public health problem, and it is envisioning zero transmission of *g*-HAT by 2030. For *r*-HAT, however, occasional cases are likely to persist beyond this point. In contrast to *T. b. gambiense*, *T. b. rhodesiense* mostly infects cattle and wild animals such as antelopes or buffalos and even carnivores. The large zoonotic reservoir is likely to be a source of infection for tsetse flies, and ultimately humans, with *T. b. rhodesiense*. Hence it will be key to the global elimination of HAT to have an uncomplicated diagnostic and treatment algorithm not only for *g*-HAT but also for *r*-HAT. This could require including acoziborole as a new, oral, single-dose regimen, if evidence supports its safety and efficacy for *r*-HAT.

Here we present a detailed in vitro evaluation of acoziborole against all three *T. brucei* subspecies regarding its efficacy, time-to-kill relationship, drug reversibility effect, and pharmacodynamics. The aim is to strengthen the preclinical dossier of acoziborole and pave the way for the extension of its use against *r*-HAT.

## Methods

### Parasite strains and culture

*T. b. rhodesiense* strain STIB900 is a derivative of STIB704, isolated from a patient in Ifakara, Tanzania, in 1982 [17]. *T. b. rhodesiense* STIB847 is a derivative of the strain UTRO 211190, isolated from a patient in Busoga, Uganda, in 1990 [17]. *T. b. rhodesiense* STIB871 is a derivative of the multidrug-resistant strain UTRO 170694, isolated from a patient in south-eastern Uganda in 1994 [18]. *T. b. rhodesiense* STIB879 is a derivative of the KETRI 2538 strain, isolated from a patient in Tete Province, Mozambique, in 1980 [19]. *T. b. gambiense* strain STIB930 is a derivate of the TH1/78E(031) strain isolated from a patient in Côte d’Ivoire in 1978 [20]. *T. b. brucei* strain STIB795 is a derivative of the S427 strain isolated in Uganda in 1960 [21]. The derivative had been received from S. Black, ILRAD Nairobi, in 1988 from continuous culture in [22] with heat-inactivated fetal bovine serum (iFBS). It was then renamed, cultured, and cryopreserved as STIB795. In 2003 and 2016 it went through a mouse passage, was adapted to axenic culture, and subsequently cryopreserved.

All trypanosomes were propagated as bloodstream forms. The *T. b. rhodesiense* and *T. b. brucei* strains were cultured at 37 °C under a humidified 5% CO_2_ atmosphere in slightly modified version of HMI-9 medium [23], consisting of Iscove's modification of Dulbecco's medium (IMDM, Sigma Aldrich) plus 3.3 mM glutamine, 0.2 mM 2-mercaptoethanol, 1 mM Na pyruvate, 1 mM hypoxanthine, 50 µM bathocuproine sulfate, 1.5 mM L-cysteine, and 160 µM thymidine. The medium was supplemented with 15% heat-inactivated horse serum for *T. b. brucei* and *T. b. rhodesiense*; for *T. b. gambiense*, the medium was supplemented with 15% iFBS and 5% human serum. To ensure maintenance in the exponential growth phase, parasites were sub-passaged into fresh medium at appropriate dilutions every 2–3 days. The population doubling times for these cultures were 12.9 ± 1.1 h for *T. b. rhodesiense* STIB900, 15.3 ± 2.7 h for *T. b. gambiense* STIB930, and 9.8 ± 0.1 h for *T. b. brucei* STIB795.

### Drug susceptibility assays

The 50% inhibitory concentrations (IC_50_) were determined with the Alamar blue assay [24]. Serial drug dilutions were prepared in 96-well microtiter plates containing the appropriate culture medium for each *T. brucei* subspecies as described above. The wells were inoculated with bloodstream-form trypanosomes: 2 × 10^3^ for *T. b. rhodesiense* and *T. b. brucei*, 5 × 10^3^ for *T. b. gambiense*. The cultures were incubated at 37 °C under a humidified 5% CO_2_ atmosphere for 70 h. Then, 10 µl of resazurin solution (12.5 mg resazurin [Sigma] dissolved in 100 ml PBS) were added to each well. The plates were incubated for an additional 2–4 h for *T. b. rhodesiense* and *T. b. brucei*, or an additional 6–8 h for *T. b. gambiense*. The plates were read in a Spectramax Gemini EM microplate fluorescence scanner (Molecular Devices) at 536 nm excitation and 588 nm emission. The IC_50_ and IC_90_ values were calculated by 4-parameter logistic regression from the sigmoidal dose-inhibition curves using GraphPad Prism 8.2.1. Assays were completed in three independent biological replicates.

### Time-kill assays

The time-dependence required by compounds to complete parasite killing was investigated at different concentrations and exposure times using the CellTiter Glo kit (Promega) to measure ATP content as a real-time indicator of viability. Test compounds were serially diluted (2-fold steps) from 100 to 0.1 µg/ml into white wall, clear bottom 96-well plates containing appropriate culture medium as described above for each parasite strain. Bloodstream-form trypanosomes were added to each well, 1 × 10^4^ for *T. b. rhodesiense* or *T. b. brucei* and 2 × 10^4^ for *T. b. gambiense*. At 2, 4, 6, 8, 12, 15, 18, and 24 h of exposure, the CellTiter Glo reagent was added to lyse the parasites, mixed for 2 min, and then incubated for 10 min in the dark. Luminescence was quantified using a Tecan Spark plate reader. Time-kill parameters were determined from plots of parasite viability versus incubation time for each concentration tested. The IC_50_ and IC_90_ values were calculated by 4-parameter logistic regression from the sigmoidal dose-inhibition curves using GraphPad Prism 8.2.1. Assays were completed in two independent biological replicates.

### Reversibility assays

The time and concentration required to induce an irreversible effect of the compound were investigated by measuring viability after transient drug exposure followed by a recovery period of 72 hours. Test compounds were serially diluted (2-fold steps) from 100 to 0.1 µg/ml into 96-well V-bottom plates containing appropriate culture medium as described above for each parasite strain. 10^4^ bloodstream forms for *T. b. rhodesiense* or *T. b. brucei,* or 2 × 10^4^ trypanosomes for *T. b. gambiense*, were added to each well. At 2, 4, 6, 8, 12, 15, 18, and 24 h exposure, the plates were centrifuged at 2000 RCF for 3 minutes to sediment the parasites. 50 µl of the supernatant was aspirated from each well and 200 µl corresponding media was added to each well. The plates were centrifuged again, 200 µl supernatant was aspirated and 200 µl corresponding media was added to each well. This wash step was repeated three more times. After the final wash, 200 µl supernatant was removed and 50 µl medium was added to achieve a final parasite suspension of 100 µl per well. 20 µl of this suspension was added to 80 µl culture medium in triplicate plates. Following 70 h incubation at 37 °C under a humidified 5% CO_2_ atmosphere, resazurin was added and trypanocidal activity was determined as described in the in vitro sensitivity assay. The IC_50_ and IC_90_ values were calculated by 4-parameter logistic regression from the sigmoidal dose-inhibition curves using GraphPad Prism 8.2.1. Assays were completed in two independent biological replicates, each run in triplicate.

### Isothermal microcalorimetry assays

Isothermal microcalorimetry with *T. b. rhodesiense* bloodstream forms was performed in a TAM III calorimeter (TA Instruments) as described [25,26]. In brief, prewarmed trypanosome suspensions and drug solutions were mixed to a final density of 10^5^ cells/ml and final concentrations of 3 × , 10 × , and 30 × the IC_50_ in a total volume of 2 ml, all in duplicate. The heat flow (µW) was continuously measured at 37 °C for up to 7 days. The recorded data were reduced to one data point per 90 s and exported to Microsoft Excel for graphics and to R for calculation of the pharmacodynamic parameters [25].

## Results

### In vitro drug sensitivity

As a first test to compare the sensitivities of the three *T. brucei* subspecies to acoziborole, the corresponding IC_50_ and IC_90_ values were determined in a standard serial drug dilution assay [24]. Acoziborole was tested against four different *T. b. rhodesiense* strains (STIB900, STIB847, STIB871, STIB879) plus *T. b. gambiense* STIB930 and *T. b. brucei* STIB795 as a reference. The inhibition curves are shown in Fig 1, and the determined IC_50_ and IC_90_ values are summarized in Table 1. The IC_50_ ranged from 0.25 to 0.64 µM, comparable to the published values of 0.18 to 0.99 µM [9]. *T. b. brucei* STIB795, which was the fastest growing of the tested strains (see Methods), was slightly more sensitive to acoziborole than the *T. b. rhodesiense* and *gambiense* strains. These latter two subspecies showed very similar drug susceptibilities to acoziborole. There were no significant differences between the four investigated *T. b. rhodesiense* strains, in spite of the fact that one of them (STIB871) was a naturally multidrug-resistant isolate [18]. All the tested strains exhibited a steep dose-response curve (Fig 1). For all subsequent more elaborate in vitro tests, we concentrated on the three strains *T. b. rhodesiense* STIB900, *T. b. gambiense* STIB930, and *T. b. brucei* STIB795.

**Table 1:** In vitro activity of acoziborole as determined with a standard Alamar blue assay. All values are in µM ± standard deviation (n = 3). Melarsoprol was included as a reference drug.

		Acoziborole		Melarsoprol	
**Subspecies**	**Strain**	**IC**_**50**_	**IC**_**90**_	**IC**_**50**_	**IC**_**90**_
*T. b. rhodesiense*	STIB900	0.57 ± 0.058	0.80 ± 0.182	0.008 ± 0.001	0.014 ± 0.003
	STIB847	0.51 ± 0.105	0.88 ± 0.080	0.003 ± 0.0004	0.008 ± 0.005
	STIB871	0.64 ± 0.070	0.86 ± 0.043	0.008 ± 0.0002	0.018 ± 0.003
	STIB879	0.62 ± 0.076	0.82 ± 0.106	0.010 ± 0.001	0.014 ± 0.001
*T. b. gambiense*	STIB930	0.63 ± 0.043	0.92 ± 0.072	0.01 ± 0.006	0.016 ± 0.007
*T. b. brucei*	STIB795	0.25 ± 0.096	0.39 ± 0.213	0.011 ± 0.002	0.015 ± 0.002

**Fig 1:**
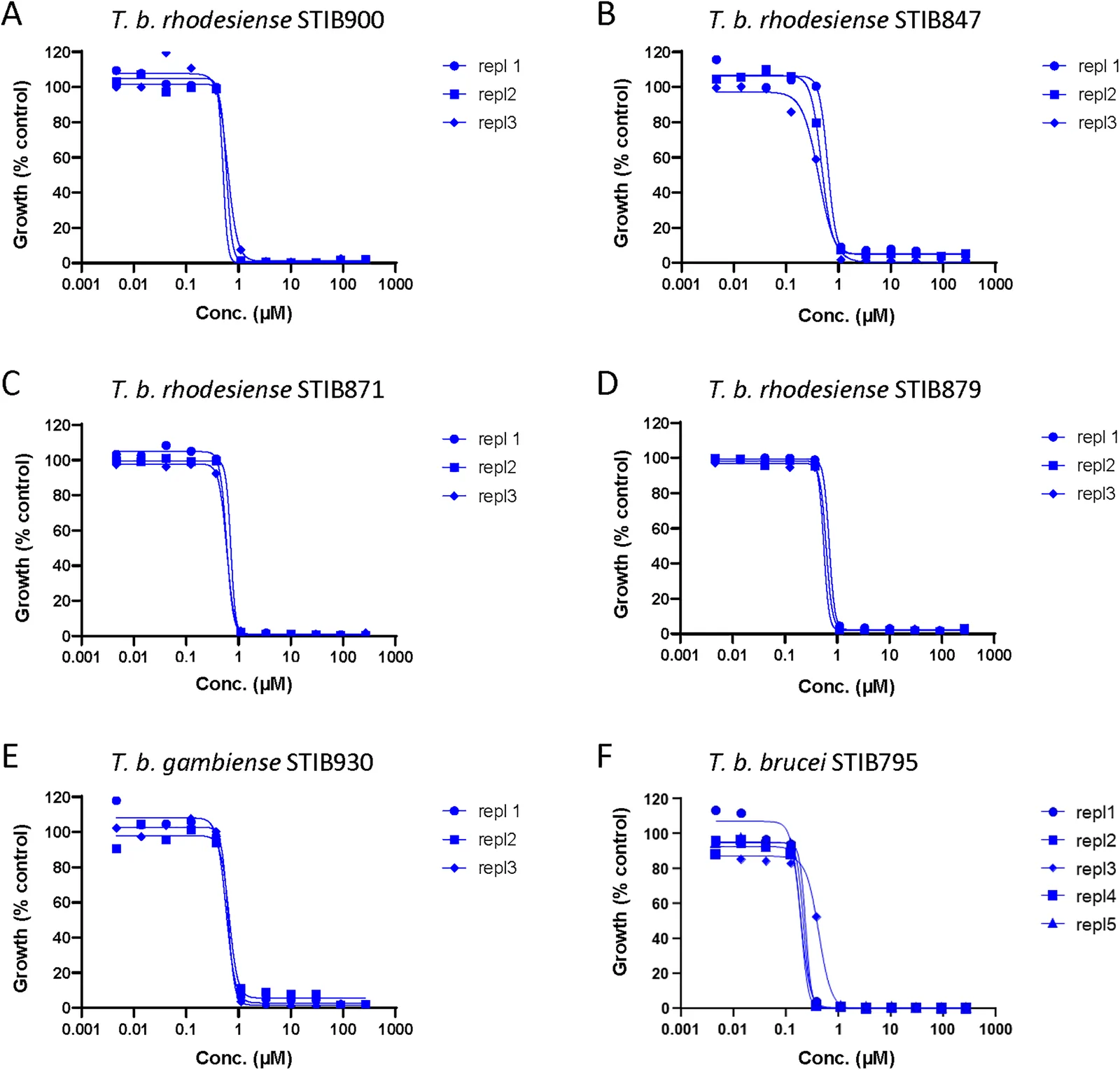
Dose-response curves for acoziborole tested against *T. b. rhodesiense* STIB900 (A), *T. b. rhodesiense* STIB847 (B), *T. b. rhodesiense* STIB871 (C), *T. b. rhodesiense* STIB879 (D), *T. b. gambiense* STIB930 (E), and *T. b. brucei* STIB795 (F). Individual biological replicates are shown, each carried out in duplicate.

### Time-kill relationship

The relationship between drug incubation time and parasite killing was investigated against bloodstream-form *T. b. rhodesiense* STIB900, *T. b. gambiense* STIB930, and *T. b. brucei* STIB795 by measuring cellular ATP content as an indicator of cell viability in real time [9]. The inocula were adjusted to the growth rates (see Methods), and the cultures were monitored over 24 h. Overall, the time-kill curves for *T. b. rhodesiense*, *T. b. gambiense*, and *T. b. brucei* looked similar to those already published [9] and indicated concentration-dependent trypanocidal activity (Fig 2). Concentrations ≥4.3 μM (1.6 μg/ml) completely killed *T. b. rhodesiense* and *T. b. brucei* parasites within 18–24 h, and *T. b. gambiense* within 12 h. From the dose-response curves at the different time points (Fig 3), the corresponding IC_50_ and IC_90_ values were calculated for each incubation period (Table 2). The dose-response curves and the calculated IC_50_ and IC_90_ were very similar for the three tested strains, with a marked effect on the parasite killing observed after >12 h of drug incubation.

**Table 2:** Time-dependence of the in vitro activity of acoziborole against *T. brucei* subspecies as based on real-time monitoring of cellular ATP content. IC_50_ and IC_90_ values are in µM ± mean absolute deviation (MAD) of two independent experiments, each carried out in duplicate.

	*T. b. rhodesiense* STIB900		*T. b. gambiense* STIB930		*T. b. brucei* STIB795	
	**IC**_**50**_	**IC**_**90**_	**IC**_**50**_	**IC**_**90**_	**IC**_**50**_	**IC**_**90**_
2 h	>270	>270	>270	>270	>270	>270
4 h	>270	>270	79 ± 37	>270	>270	>270
6 h	170 ± 1.7	>270	7.0 ± 0.50	130 ± 20	182 ± 0.60	>270
8 h	9.7 ± 0.09	>270	3.6 ± 0.08	69 ± 14	40 ± 28	>270
12 h	3.5 ± 0.19	210 ± 20	1.5 ± 0.18	3.1 ± 0.22	2.3 ± 0.15	>270
15 h	1.9 ± 0.11	4.4 ± 0.35	1.5 ± 0.17	2.3 ± 0.19	1.9 ± 0.12	220 ± 14
18 h	1.5 ± 0.03	2.7 ± 0.18	1.2 ± 0.31	1.9 ± 0.19	1.6 ± 0.02	57 ± 51
24 h	1.4 ± 0.05	2.0 ± 0.06	0.80 ± 0.00	1.2 ± 0.07	1.3 ± 0.07	2.6 ± 0.59

**Fig 2:**
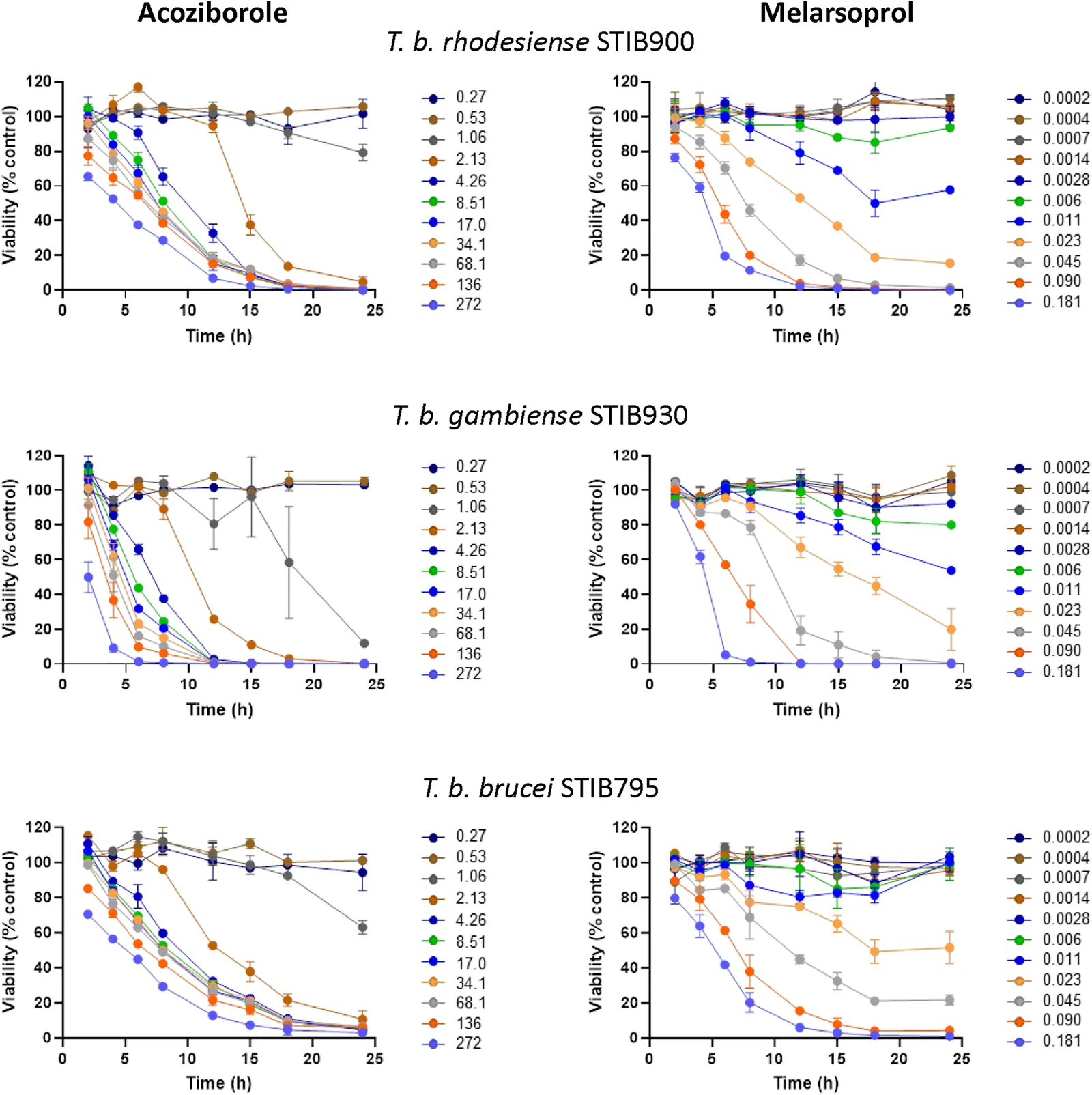
Viability following continuous exposure of *T. brucei* subspecies to acoziborole at the indicated concentrations for up to 24 h. Viability was determined by measuring ATP content using Celltiter Glo. Data are mean ±MAD (mean absolute deviation); n = 2. Melarsoprol was included as a reference drug. The different colours represent concentrations in µM.

**Fig 3:**
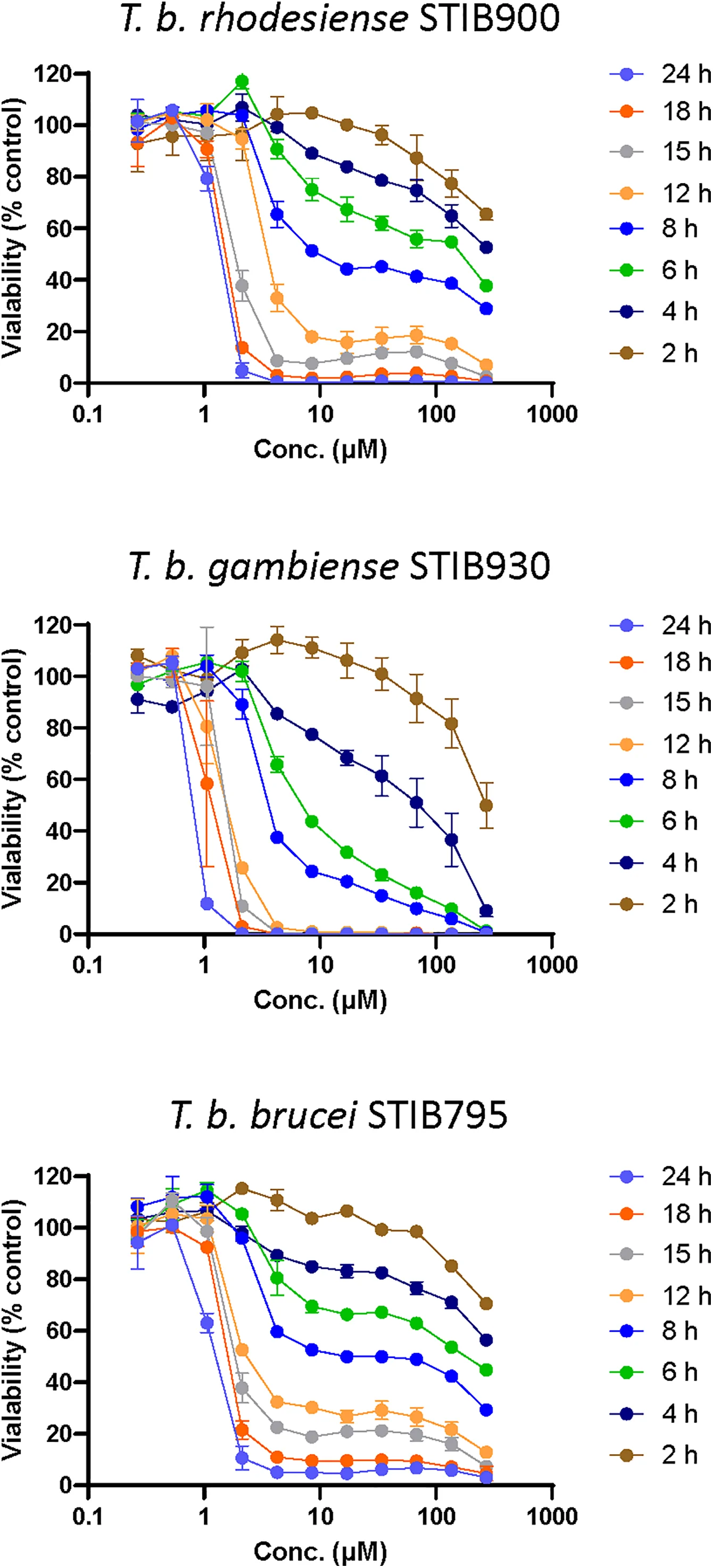
The data from Fig 2 plotted as dose-response curves for acoziborole at the different drug exposure times. Data are mean ±MAD; n = 2. The different colours represent incubation times.

### Reversibility of drug effect

The reversibility of the trypanocidal effect over time at different drug concentrations was further investigated using washout assays [26]. *T. b. rhodesiense* STIB900, *T. b. gambiense* STIB930, and *T. b. brucei* STIB795 were incubated with drug for different time periods from 2 to 24 h, thoroughly washed, and further incubated without drug for 72 h (see Methods). Acoziborole showed an irreversible effect following 24 h of drug incubation at concentrations of ≥2.1 μM (0.78 μg/ml) for *T. b. brucei* and *T. b. rhodesiense*, and ≥1.1 μM (0.39 μg/ml) for *T. b. gambiense* (Fig 4). Lower drug concentrations had a reversible drug effect in the sense that they did not kill all trypanosomes, as became apparent after drug washout and regrowth of the cultures (Fig 4). From the dose-response curves at different time points (Fig 5), the corresponding IC_50_ and IC_90_ values were calculated for each incubation period (Table 3). The dose-response curves and the IC_50_ and IC_90_ values were similar for all tested *T. brucei* subspecies at 24 h, 18 h, and 15 h of drug exposure. For shorter drug exposure periods, *T. b. rhodesiense* STIB900 exhibited a lower susceptibility to acoziborole than the *T. b. brucei* and the *T. b. gambiense* strains.

**Table 3:** Time-dependence of the *in vitro* activity of acoziborole against T. brucei subspecies as based on their recovery 72 h after drug washout. IC50 and IC90 values are in µM ± MAD of two independent experiments, each carried out in duplicate. The times indicate the duration of drug exposure prior to the washout step.

	*T. b. rhodesiense* STIB900		*T. b. gambiense* STIB930		*T. b. brucei* STIB795	
	IC_50_	IC_90_	IC_50_	IC_90_	IC_50_	IC_90_
2 h	79 ± 68	>270	14 ± 11	21 ± 14	11 ± 5.2	80 ± 31
4 h	55 ± 43	>270	12 ± 8.7	18 ± 14	3.3 ± 0.26	17 ± 5.7
6 h	62 ± 14	180 ± 48	1.2 ± 2.1	2.1 ± 0.00	2.2 ± 0.65	10 ± 4.8
8 h	12 ± 7.9	140 ± 57	1.1 ± 0.30	1.8 ± 0.19	1.5 ± 0.07	5.8 ± 1.9
12 h	4.0 ± 2.0	6.1 ± 1.9	0.78 ± 0.03	1.0 ± 0.01	1.4 ± 0.04	2.0 ± 0.02
15 h	2.3 ± 0.75	3.1 ± 1.0	0.76 ± 0.02	1.1 ± 0.07	1.4 ± 0.12	2.1 ± 0.12
18 h	1.6 ± 0.06	2.5 ± 0.45	0.83 ± 0.05	1.4 ± 0.34	1.4 ± 0.02	2.0 ± 0.01
24 h	1.5 ± 0.11	2.0 ± 0.26	0.85 ± 0.13	1.4 ± 0.43	0.80 ± 0.04	1.7 ± 0.04

**Fig 4:**
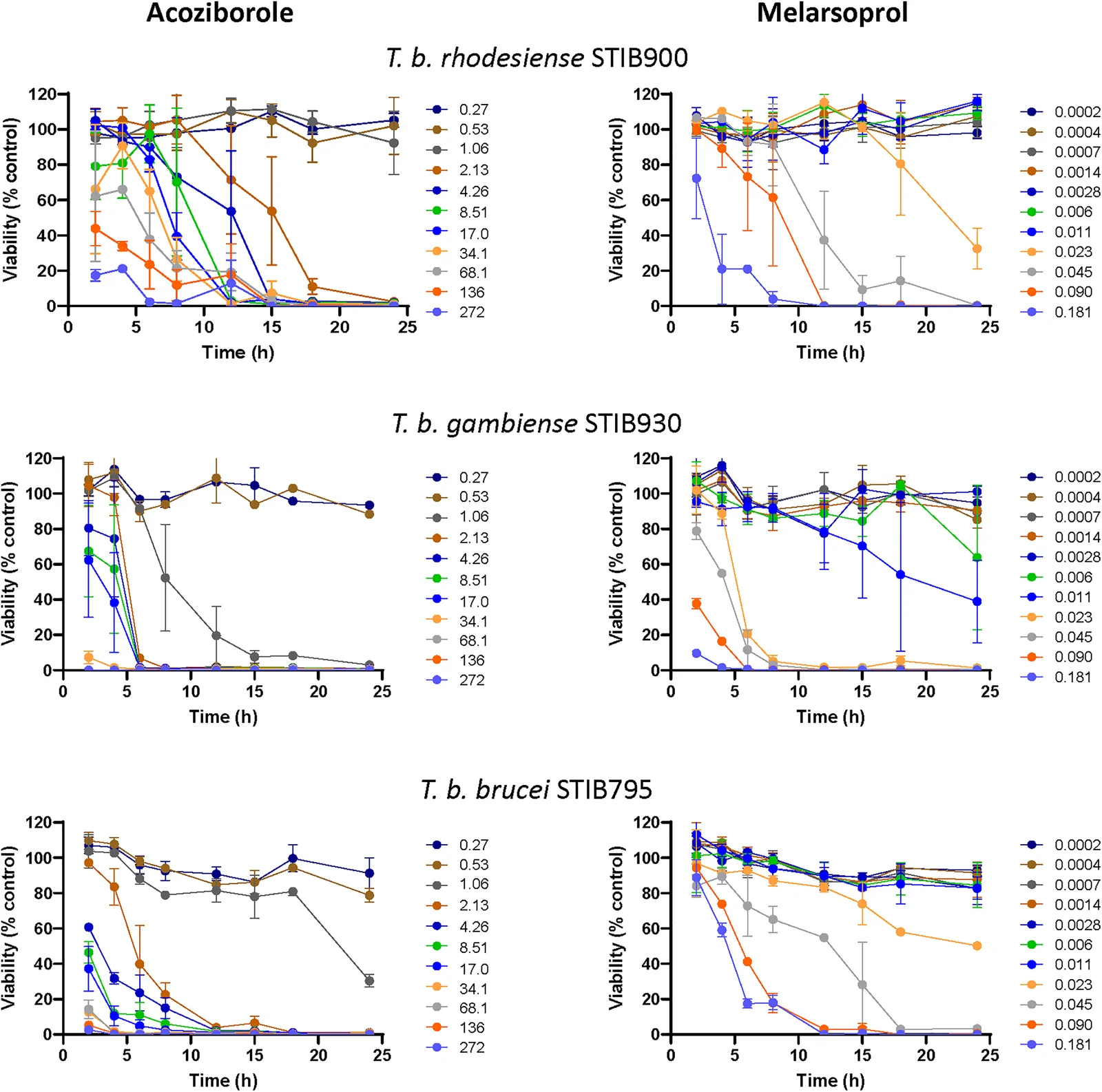
Reversibility of the antitrypanosomal effect. *T. b. rhodesiense* STIB900, *T. b. gambiense* STIB930, and *T. b. brucei* STIB795 were exposed to the indicated drug concentrations for 2, 4, 6, 8, 12, 15, 18, or 24 h; then the drug was washed out and parasite viability was determined after a further incubation for 72 h in fresh medium without drug. The different colours represent concentrations in µM.

**Fig 5:**
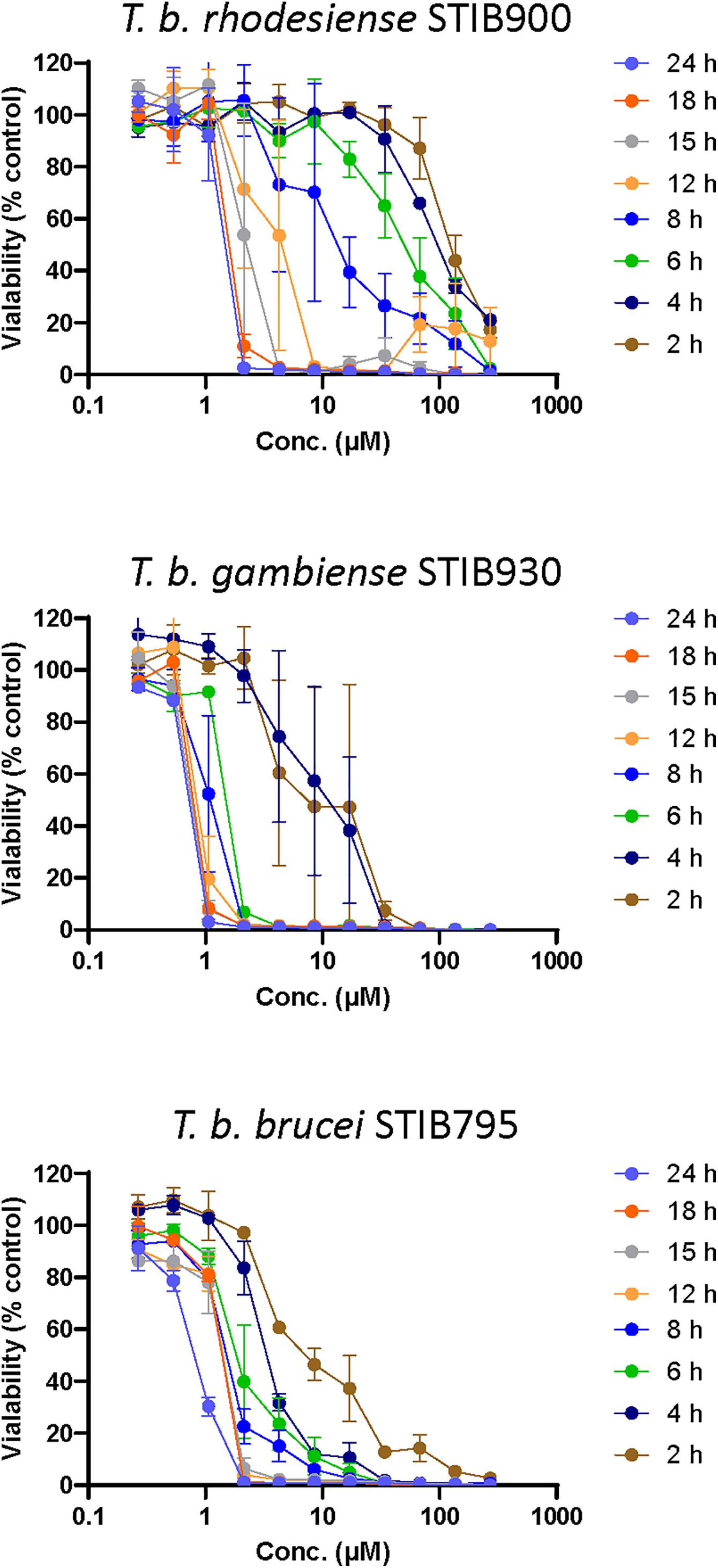
The data from Fig 4 plotted as dose-response curves for acoziborole at the different drug exposure times. Data are mean ±MAD; n = 2. The different colours represent incubation times.

### Isothermal microcalorimetry

To monitor drug action against *T. b. rhodesiense* in real time, we performed isothermal microcalorimetry using heat flow as a proxy for the number of viable parasites [27]. In a set-up that has proven itself in our laboratory, bloodstream-form cultures were exposed to acoziborole at concentrations of one to thirty times the IC_50_ value [26]. Fexinidazole and its two metabolites, fexinidazole sulfoxide and fexinidazole sulfone [1], were included for comparison at the same concentration range relative to the corresponding IC_50_. Pharmacodynamic parameters (Table 4) were determined from the heat flow curves (Fig 6) as follows: the onset of drug action was defined as the timepoint where the slopes of treated and untreated cultures diverged by more than 0.04 μW/h. Time-to-kill was defined as the timepoint where the slope of the smoothed curve flattened below 0.005 μW/h.

**Table 4:** Pharmacodynamic parameters of drug action as calculated from the corresponding heat flow curves with *T. b. rhodesiense* STIB900 (Fig 6). Drug concentrations are given in multiples of the respective IC50 for ease of reference to published data [9,26].

	Concentration [IC_50_]	Onset of action [h]	Time-to-kill [h]
Acoziborole	3×	3.4	44
Acoziborole	10×	3.5	14
Acoziborole	30×	2.5	<12
Fexinidazole	3×	6.5	116
Fexinidazole	10×	6.1	54
Fexinidazole	30×	6	50
Fexinidazole sulfoxide	3×	8.9	80
Fexinidazole sulfoxide	10×	6.4	34
Fexinidazole sulfoxide	30×	5.2	14
Fexinidazole sulfone	3×	8.8	63
Fexinidazole sulfone	10×	6.2	24
Fexinidazole sulfone	30×	4.7	24

**Fig 6:**
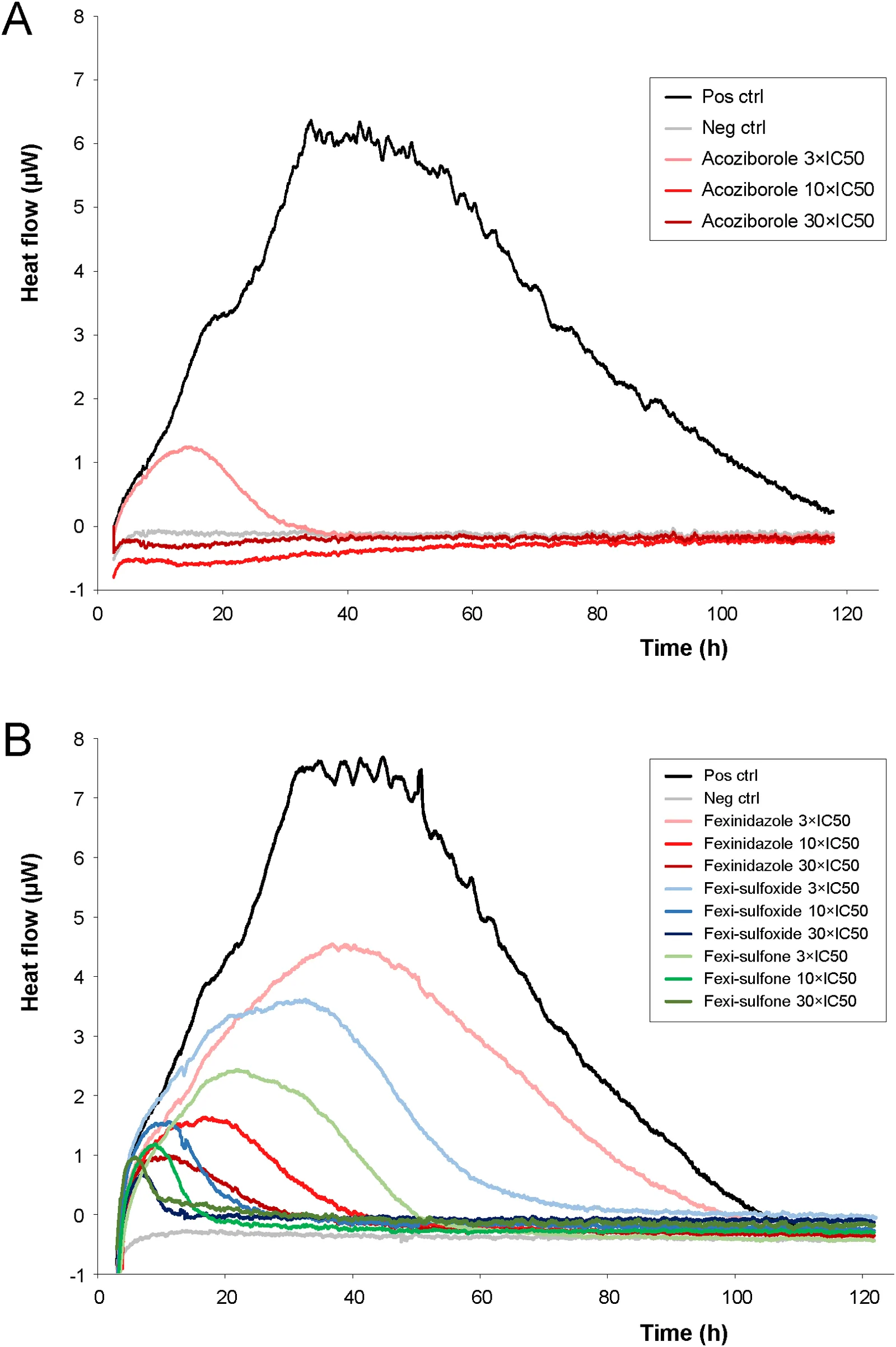
Real-time isothermal microcalorimetry with next generation trypanocides against *T. b. rhodesiense* STIB900. Mean heat flow curves (µW) of two independent experiments, each run in duplicate, for acoziborole (A) and fexinidazole plus its metabolites **(B)**. Drug concentrations are given in multiples of IC_50_ for ease of reference to published data [9,26]. The IC_50_ values were 0.2 µg/ml for acoziborole; 0.7 µg/ml for fexinidazole; 0.5 µg/ml for fexinidazole sulfoxide; 0.4 µg/ml for fexinidazole sulfone. The positive controls (black) are trypanosomes without drug; the negative controls (grey) are medium without trypanosomes.

At a concentration of three times the IC_50_ (1 µg/ml, equivalent to 2.7 µM), acoziborole had a strong effect on parasite growth compared to controls (i.e., untreated trypanosomes). This effect was observed after 3–4 h, and led to a total loss of parasite viability in less than 48 h at 3 times IC_50_ value. Higher concentrations (10 and 30 × IC_50_) of acoziborole killed the parasites in less than 16 h (Fig 6, Table 4). Acoziborole showed a significantly faster onset of action and a shorter time-to-kill effect than fexinidazole and its major metabolites (Fig 6, Table 4). At a concentration of three times the IC_50_, fexinidazole, fexinidazole sulfoxide, and fexinidazole sulfone killed the trypanosomes after 116, 80, and 63 h, respectively.

## Discussion

HAT is one of the very few infectious diseases where elimination seems tangible, at least for *g*-HAT, which does not have a relevant zoonotic reservoir. The goal of eliminating *g*-HAT as a public health problem has been reached [16], and the WHO is now envisaging zero transmission of *g*-HAT by 2030. To win the endgame also against the zoonotic *r*-HAT, acoziborole as a single-dose cure would be of tremendous help to ensure a rapid response in case of sporadic *T. b. rhodesiense* infections or outbreaks. However, cross-reactivity against both *T. b. rhodesiense* and *T. b. gambiense* cannot be taken for granted, as exemplified by the fact that the drug eflornithine is effective only against the latter [19, 28]. Although fexinidazole already has greatly improved the case management of *r*-HAT [5], the current focus is an easier treatment as well as getting rid of melarsoprol that – in spite of its high toxicity – remains the sole treatment option for severe late-stage *r*-HAT in children under six years old. To validate the potential of acoziborole for *r*-HAT, we performed in-depth in vitro preclinical profiling of the efficacy and pharmacodynamics of acoziborole in *T. b. rhodesiense*, *T. b. brucei,* and *T. b. gambiense*.

Direct comparison of the *T. brucei* subspecies in terms of drug susceptibility is not perfectly straightforward, due to differences in the culture media and growth characteristics. Bloodstream forms of *T. b. gambiense* require human serum in the medium, whereas *T. b. rhodesiense* and *T. b. brucei* grow in standard medium supplemented with fetal calf serum or horse serum (human serum is lethal to *T. b. brucei*). Also the growth rates differ, as do the maximal densities obtained in culture. *T. b. gambiense* has the lowest cell density and has the highest population doubling time, reflecting the clinical presentation of *g*-HAT, which progresses more slowly than *r*-HAT, and has a lower blood parasitemia [29,30]. Taking into account these differences by adapting the inocula, we determined the drug susceptibility of four *T. b. rhodesiense* strains obtained from different regions (in Tanzania, Uganda, Mozambique) and compared them with *T. b. brucei* (from Uganda) and *T. b. gambiense* (from Côte d’Ivoire) strains. Acoziborole exhibited very similar efficacy against all the tested strains, with very steep dose-response curves and IC_50_ values in agreement with the published data [9]. In particular, there was hardly any difference between the four *T. b. rhodesiense* strains, with IC_50_ values in a narrow window around 600 nM. The different *T. b. rhodesiense* strains were somewhat less uniform, although still consistently very tight in their drug susceptibility to melarsoprol, with IC_50_ ranging from 3 nM to 11 nM.

The pharmacodynamics of acoziborole were assessed with three in vitro assay systems: time-to-kill was first determined via the decrease of cellular ATP levels during drug exposure. The reversibility of drug action was assessed using a washout protocol, in which the parasites were exposed to the drug for different time windows, then washed, and further incubated for 72 h to give the surviving trypanosomes a chance to recover. Finally, isothermal microcalorimetry was used as a highly sensitive and non-invasive tool to monitor onset of drug action and time-to-kill. Given the very consistently tight drug susceptibilities obtained with acoziborole in the four different *T. b. rhodesiense* strains, these more laborious drug action assays were carried out with one strain of *T. b. rhodesiense* (STIB900) and compared with *T. b. brucei* (STIB795) and *T. b. gambiense* (STIB930).

The results described above are very similar to the values used to define the PK/PD driver of efficacy of acoziborole and, notably, the concentration of the drug required to achieve cure of the chronic (stage 2) *T. b. brucei* murine model [9,31]. More specifically, the time-kill curves of acoziborole against *T. b. rhodesiense* were practically identical to those previously published for *T. b. brucei* and reached MIC values of 0.78 µg/ml, closely matching the published MIC of 0.63 µg/ml [9]. Additionally, acoziborole concentrations of ≥2.1 µM (0.78 µg/ml) over a 24 h drug incubation period resulted in complete parasite killing for all *T. brucei* subspecies. At shorter incubation times (6–15 h), *T. b. gambiense* and *T. b. brucei* were being killed slightly more readily than *T. b. rhodesiense*. Previously published reversibility assays conducted with acoziborole (SCYX-7158) indicated that exposure time and AUC/MIC were important in predicting efficacy and that a minimum inhibitory concentration (MIC) of 0.6 µg/ml was necessary to complete full clearance of parasites in vitro at the 24 h timepoint [9]. This MIC corresponds to an unbound AUC_0–24_ of 5.81 µg·h/ml that was proven necessary to address the chronic stage of the infection and cure the *T. b. brucei* murine model [31]. Such MIC values have been reached in the reversibility studies completed as part of this study for all *T. brucei* subspecies, indicating that *T. b. rhodesiense* behaves very similarly to *T. b. brucei* and *T. b. gambiense* with regard to drug susceptibility and pharmacodynamics.

Acoziborole is administered to HAT patients orally as a single dose of 960 mg (three tables of 320 mg) [10]. The drug appears in plasma at 1 h, reaches maximal levels between 24 h and 72 h, which remain stable for up to 96 h and then decrease with a half-life of around 400 h [12]. A study in human volunteers receiving a microtracer dose of radiolabeled acoziborole confirmed the good oral absorption of acoziborole, and it demonstrated a very limited metabolism and very slow biliary-fecal elimination [32]. The long half-life of elimination of acoziborole ensures that sustained levels of the drug are present in the target tissues allowing complete parasite killing over time [12]. Complementary isothermal microcalorimetry studies showed a fast onset of action for acoziborole after only about 3 h of drug incubation, in line with the initiation of the killing observed in time-to-kill and reversibility studies. This was clearly shorter than for fexinidazole and its major metabolites, fexinidazole sulfoxide and fexinidazole sulfone.

In summary, acoziborole has been proven to display very similar drug susceptibility across *T. brucei* subspecies including in a small panel of *T. b. rhodesiense* strains from different regions. Similar drug action profiles of acoziborole have been observed between *T. b. brucei, T. b. rhodesiense,* and *T. b. gambiense* using time-to-kill and reversibility studies. These results match very well with results previously reported in the literature for the same assay systems and led to the same MIC values identified as being predictive of efficacy in a preclinical murine model addressing the second stage of the disease. Acoziborole was also shown to act faster than fexinidazole and its metabolites in isothermal microcalorimetry studies. The finding that acoziborole seems to kill *T. b. gambiense* slightly faster than *T. b. rhodesiense* in time-to-kill and reversibility assays, appears to be irrelevant for the treatment of patients, due to the very long half-life of elimination of acoziborole in humans (around 400 h) [12]. Based on these results, acoziborole is anticipated to demonstrate equivalent efficacy in the treatment of *r*-HAT as it does for *g*-HAT: the dosing regimen currently proposed for *T. b. gambiense* will also be sufficient to cure infections with *T. b. rhodesiense*.
